# Supplementary material for: Imprinted lncRNA Dio3os preprograms intergenerational brown fat development and obesity resistance
Source: Nat Commun. 2021 Nov 25;12:6845. doi: 10.1038/s41467-021-27171-1 (PMC8617289; doi:10.1038/s41467-021-27171-1)
Supplement: Supplementary file 1 — Supplementary Information [file 41467_2021_27171_MOESM1_ESM.pdf]

Imprinted lncRNA Dio3os preprograms intergenerational brown fat development and obesity resistance

Yan-Ting Chen, Qi-Yuan Yang, Yun Hu, Xiang-Dong Liu, Jeanene M. de Avila, Mei-Jun Zhu, Peter W. Nathanielsz, Min Du

Supplementary Information.

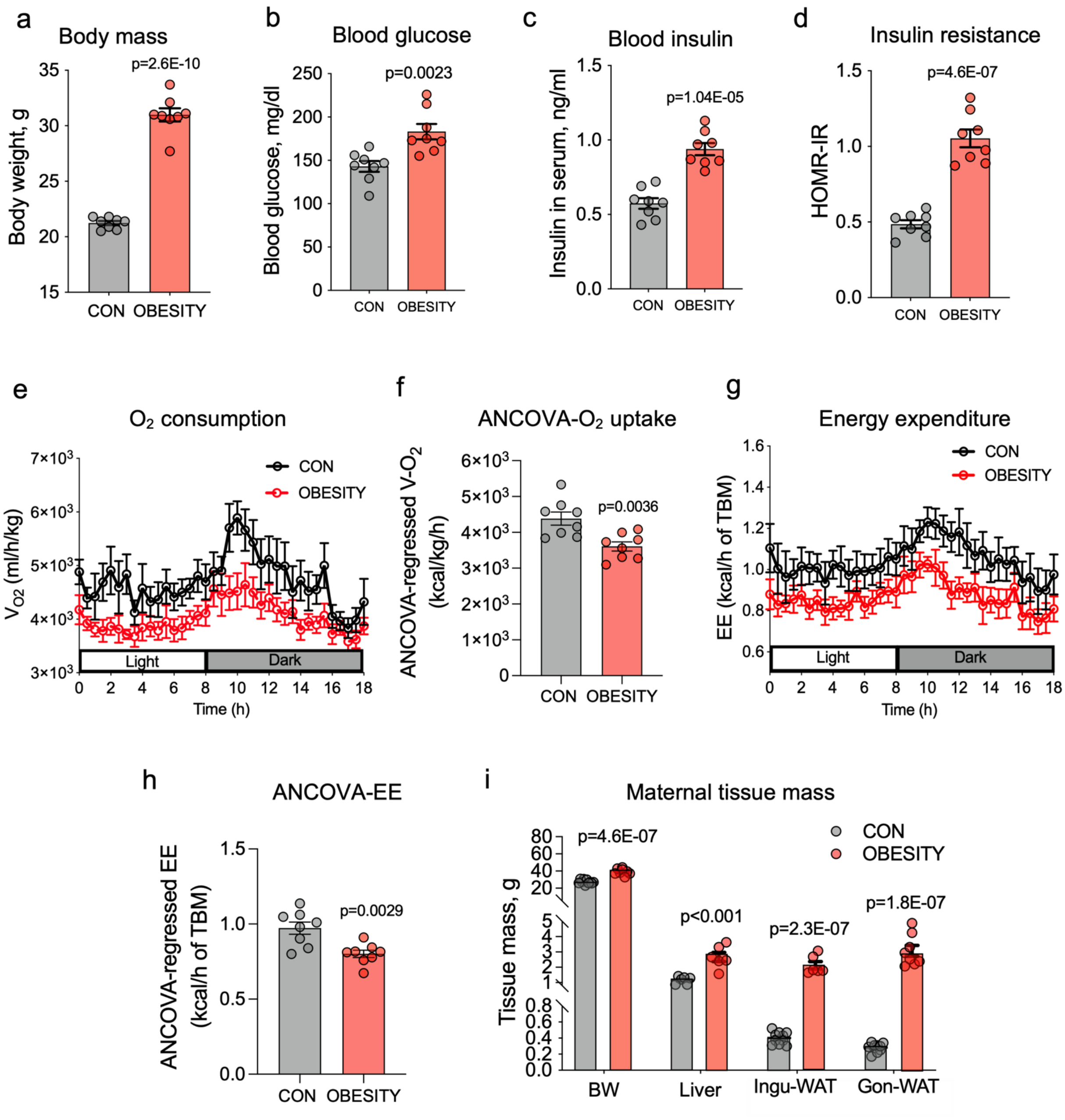

Supplementary Figure 1: Female mice fed high fat diet become obese before mating.

a-d. After 10-weeks on high fat diet (OBESITY; 45% calorie from fat) or regular diet (CON; 10% calorie from fat), measurement of body mass (a), fasting blood glucose (b), insulin (c) and insulin resistance (HOMR-IR) (d) in dam before mating (n = 8). **e-h**. Normalized O_2 consumption (e) and energy expenditure (g) in control and obese dam (n = 8). O_2 consumption and energy expenditure were regressed to total body mass (TBM) according to NIDDK MMPC

ANCOVA analyses (f and h). **i.** Body weight (*n* = 8) and tissue mass of dam at the end of lactation. Data are presented as mean ± SEM. Unpaired Student's *t*-test with two-tailed distribution was used in data analyses.

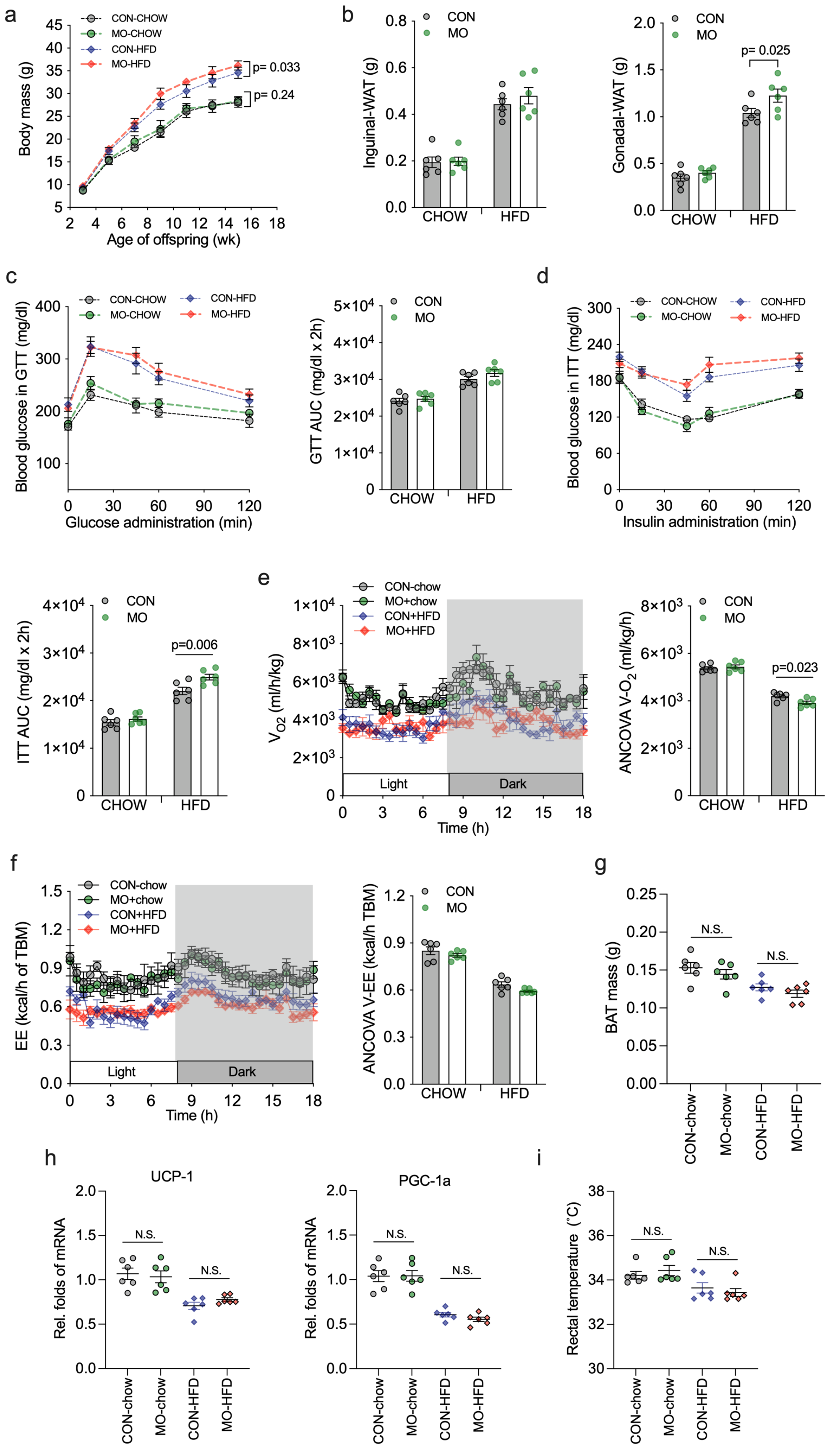

a
CON-CHOW
MO-CHOW
CON-HFD
MO-HFD
p= 0.033
p= 0.24
Body mass (g)
Age of offspring (wk)
b
CON
MO
Inguinal-WAT (g)
CHOW
HFD
Gonadal-WAT (g)
p= 0.025
c
Blood glucose in GTT (mg/dl)
Glucose administration (min)
GTT AUC (mg/dl x 2h)
d
Blood glucose in ITT (mg/dl)
Insulin administration (min)
ITT AUC (mg/dl x 2h)
p=0.006
e
CON-chow
MO+chow
CON+HFD
MO+HFD
VO2 (ml/h/kg)
Light
Dark
Time (h)
ANCOVA V-O2 (ml/kg/h)
p=0.023
f
EE (kcal/h of TBM)
ANCOVA V-EE (kcal/h TBM)
g
BAT mass (g)
N.S.
CON-chow
MO-chow
CON-HFD
MO-HFD
h
UCP-1
PGC-1a
Rel. folds of mRNA
i
Rectal temperature (˚C)

Supplementary Figure 2. Maternal obesity (MO) has few impacts on male offspring energy expenditure and brown fat thermogenesis.

a. Body weight growth of male offspring (C57BL/6J) fed normal and high fat diet (HFD) for 12-wk after weaning at 22˚C (*n* = 6). Offspring were born from normal or MO dams. **b.** Inguinal (subcutaneous-fat) and gonadal (visceral-fat) white fat mass in male offspring at 4-month-old (*n* = 6). **c, d.** Measuring blood glucose after glucose or insulin administration in male offspring for glucose tolerance (GTT) and insulin sensitivity tests (ITT) (*n* = 6). **e, f.** Measurement of oxygen consumption and energy expenditure (EE) in 4-month-old male offspring fed chow or HFD (*n* = 6). Metabolic data were regressed to total body mass (TBM) according to guideline of ANCOVA NIDDK MMPC energy expenditure tool. **g.** Brown fat mass in male offspring fed chow or HFD (*n* = 6). **h.** *Ucp-1* and *Ppargc-1a* gene expression in male offspring BAT (*n* = 6). Gene expression was normalized to 18S rRNA. **i.** Rectal temperature in male offspring (*n* = 6). Each pregnancy (dam) was used as an experimental unit. Data are presented as mean ± s.e.m. Two-way ANOVA with Bonferroni post hoc analysis was used for data analyses; body mass curve was determined by Two-way ANOVA repeated measurements.

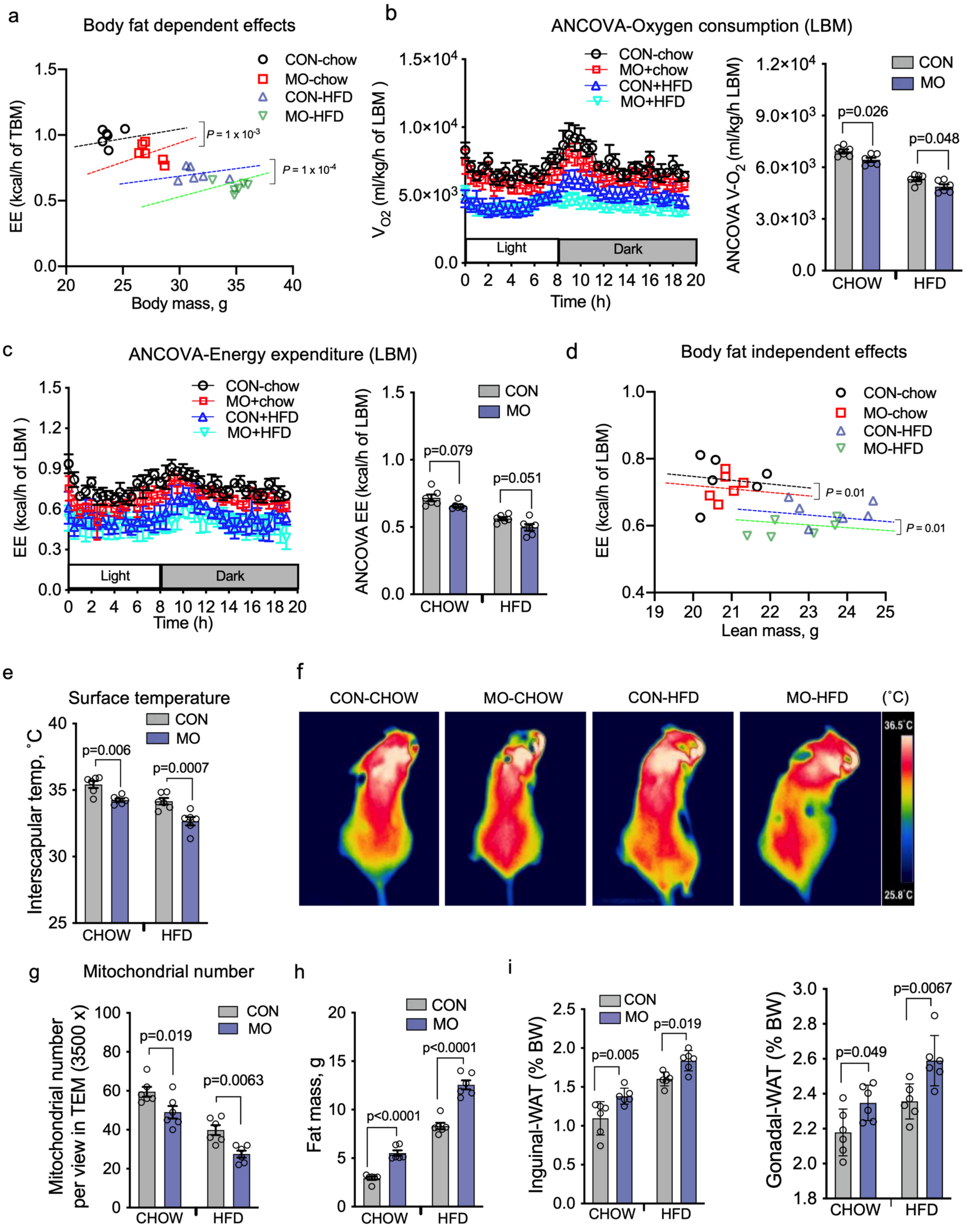

Supplementary Figure 3. Maternal obesity (MO) reduces thermogenesis and energy expenditure in female offspring.

a-d. Oxygen consumption (b) and energy expenditure (c) in female offspring at 22˚C (*n* = 6). Metabolic data were regressed to total body mass (a, TBM, fat dependent) or lean body mass (c, LBM, fat independent) according to guideline of NIDDK MMPC energy expenditure tool. **e, f.** Interscapular surface temperature of female offspring at 22˚C (*n* = 6). Thermal images were obtained after mice were immediately removed from cages to avoid heat loss. Animal behavior and scanning distance were also controlled during image capturing. **g.** Quantified mitochondrial number in female offspring BAT (*n* = 6; individual animals were taken at least 8 microscopic views). **h.** Fat mass in female offspring (*n* = 6). **i.** Mass of inguinal (sub-WAT) and gonadal (visceral-WAT) white fat (% body mass) in female offspring (*n* = 6). Data are presented as mean ± s.e.m. Each pregnancy (dam) was used as an experimental unit. Two-way ANOVA with Bonferroni post hoc analysis was used for data analyses. Metabolic data were analyzed by ANCOVA multiple regression models.

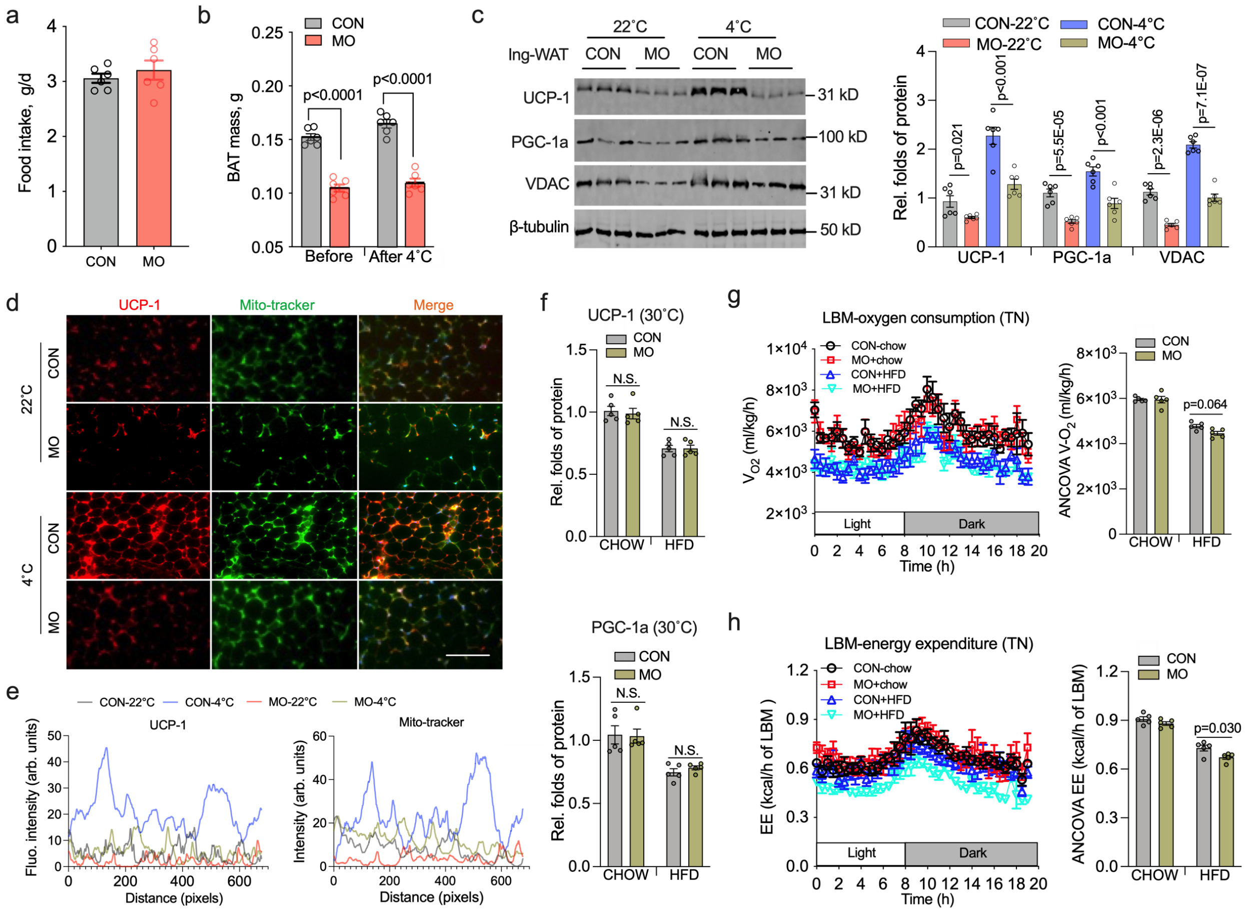

Supplementary Figure 4. Maternal obesity impairs brown adipogenesis and energy expenditure in female offspring under cold exposure.

a, b. Food intake (a) and brown fat mass (b) in female offspring fed chow diet at 4˚C (n = 6). **c**. Immunoblotting measuring UCP-1, PGC-1a and VDAC proteins in female offspring inguinal-WAT after 4˚C exposure. β-tubulin was as loading control (n = 6). **d, e.** Immunostaining of UCP-1 (red) and mitochondria (green) in inguinal-WAT after 4˚C exposure (d). Intensity was quantified by image-J (e) (n = 4). Scale bar 200 μm. **f.** Quantified protein abundance of UCP-1 and PGC-1a in female offspring BAT at 30˚C (n = 5). **g, h.** Oxygen consumption (g) and energy expenditure (h) in female offspring fed chow or high fat diet (HFD) at 30˚C (n = 5). Data were regressed to lean body mass (LBM) according to guideline of ANCOVA NIDDK MMPC energy expenditure tool. Each pregnancy (dam) was considered as a replicate unit. Data are presented as

mean ± s.e.m. Two-way ANOVA with Bonferroni post hoc analysis, and unpaired Student's *t*-test with two-tailed distribution were used in data analyses.

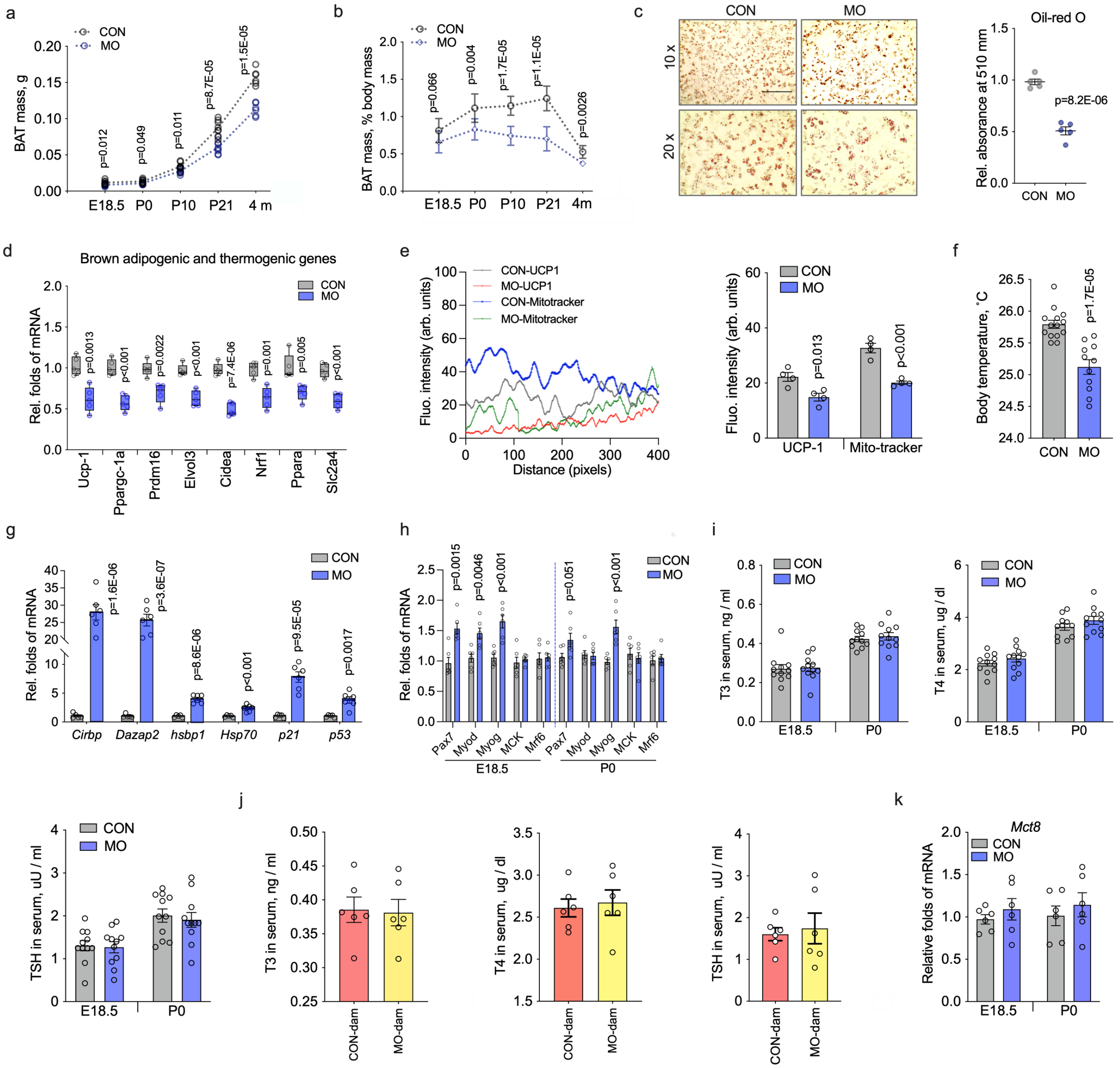

Supplementary Figure 5. Maternal obesity impairs fetal and neonatal BAT thermogenesis, increasing hypothermia risks.

a, b. BAT mass (a) and % of body mass (b) of female offspring born to control or maternal obesity (MO) dam at 22˚C (*n* = 8 at E18.5, P0 and P21; n = 6 at 4 months). **c.** Isolated stromal vascular fractions (SVFs) in fetal BAT were differentiated into brown adipocytes *in vitro*. Lipids in matured brown adipocytes were stained by Oil Red O and quantified at 510 nm (*n* = 5). Scar bar 500 μm. **d.** mRNA expression of brown adipogenic and thermogenic genes in mature brown

adipocytes differentiated from SVFs isolated from control and MO fetal BAT at E18.5. Gene expression was normalized to 18S rRNA (n = 5). Whisker of box plots shows mean and individual values from minimum to maximum. **e.** Quantified immunostaining intensity of UCP-1 and mitochondria in offspring BAT (n = 4). **f.** Quantified body temperature of female neonates (n = 14 in control; n = 11 in MO). **g.** mRNA expression of cold stress genes in dorsal skin of female neonates. mRNA expression was normalized to 18S rRNA (n = 5 in control; n = 6 in MO). **h.** mRNA expression of myogenic regulatory genes in offspring BAT at E18.5 and P0 (n = 6). Expression was normalized to 18S rRNA. **i.** T3, T4 and TSH concentrations in serum of female fetuses (n = 11). **j.** T3, T4 and TSH concentrations in serum of dam (n = 6). **k.** mRNA expression of *Mct8* in BAT of female fetuses. Gene expression was normalized to 18S RNA (n = 6). Data are presented as mean ± s.e.m. Unpaired Student's t-test with two-tailed distribution was used in data analyses.

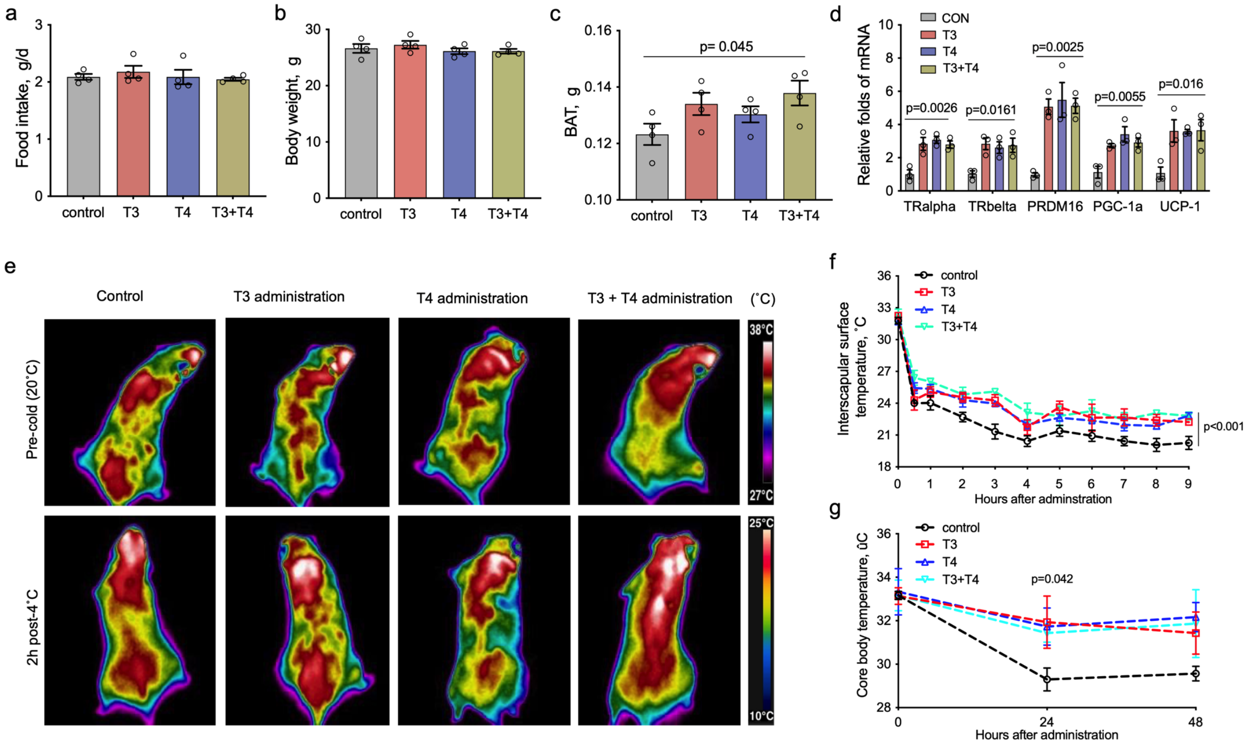

Supplementary Figure 6. Thyroid hormone activates brown adipogenesis and thermogenic responsiveness to cold exposure.

a-c. C57BL/6J mice at 4-month-old were administrated with PBS (control), 60 µg/kg T3, 180 µg/kg T4 and combination of T3 and T4 for 3 days at 4˚C. Food intake (a), body weight (b) and BAT mass (c) were measured in mice after cold exposure (n = 4). **d.** mRNA expression of genes after cold exposure. Gene expression was normalized to 18S rRNA (n = 3). **e, f.** Interscapular surface temperature of mice treated with thyroid hormones under cold exposure (n = 3). Animal behavior and scanning distance were controlled during image capturing. **g.** Core body temperature was measured in rectum using a precise digital thermal meter (n = 3). Data are presented as mean ± s.e.m. One-way ANOVA with adjusted multiple-comparison was used in data analyses.

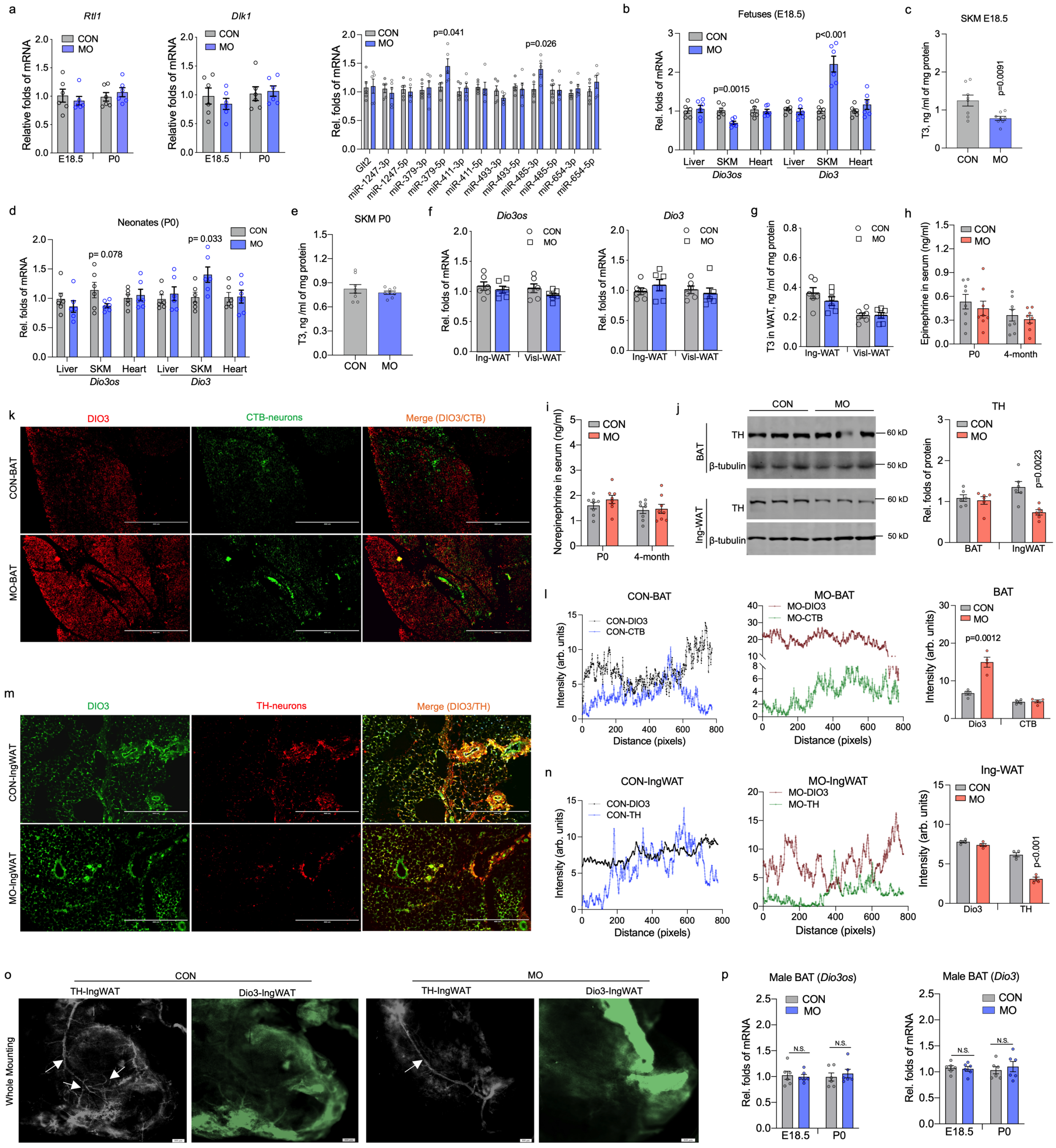

Supplementary Figure 7. Maternal obesity reduces T3 concentration in BAT of female fetuses without altering thyroid hormone concentrations in maternal-offspring blood circulation.

a. mRNA expression of imprinted genes (n = 5) and miRNAs (n = 6) in female fetal and neonatal BAT. Gene expression was normalized to 18S rRNA, and miRNA expression was normalized to U6. **b.** mRNA expression of *Dio3* and *Dio3os* in liver, limb skeletal muscle (SKM) and heart of female fetuses (n = 6). **c.** T3 content in SKM of female fetuses (n = 8). **d.** mRNA expression of *Dio3* and *Dio3os* in liver, limb SKM and heart of female neonates (n = 6). **e.** T3 content in SKM of female neonates (n = 8). **f, g.** mRNA expression of *Dio3*, *Dio3os* (f) and T3 content (g) in inguinal- and gonadal-WAT of female offspring at weaning (n = 6). **h, i.** Concentration of epinephrine (h) and non-epinephrine (i) in female offspring serum at P0 and 4-month-old (n = 8). **j.** Immunoblotting measuring neural density marked by tyrosine hydroxylase (TH) protein in neonatal offspring BAT and offspring inguinal-WAT after weaning (n = 6). **k-n.** Co-immunostaining of Dio3 with cholera toxin B (CTB) or TH in offspring BAT (P0) and inguinal-WAT (P21) (k, m). Intensity was quantified by image-J (l, n) (n = 4). Scale bar 400 µm. **o.** Whole-mounting immunostaining of TH and Dio3 in offspring inguinal-WAT (n = 4). White arrow shows neural circuits in WAT. Scale bar 800 µm. **p**. mRNA expression of *Dio3os* and *Dio3* in male fetal and neonatal BAT (n = 6). In data analyses, each pregnancy (dam) was considered as a replicate unit. Data are presented as mean ± s.e.m. Unpaired Student's *t*-test with two-tailed distribution was used in data analyses.

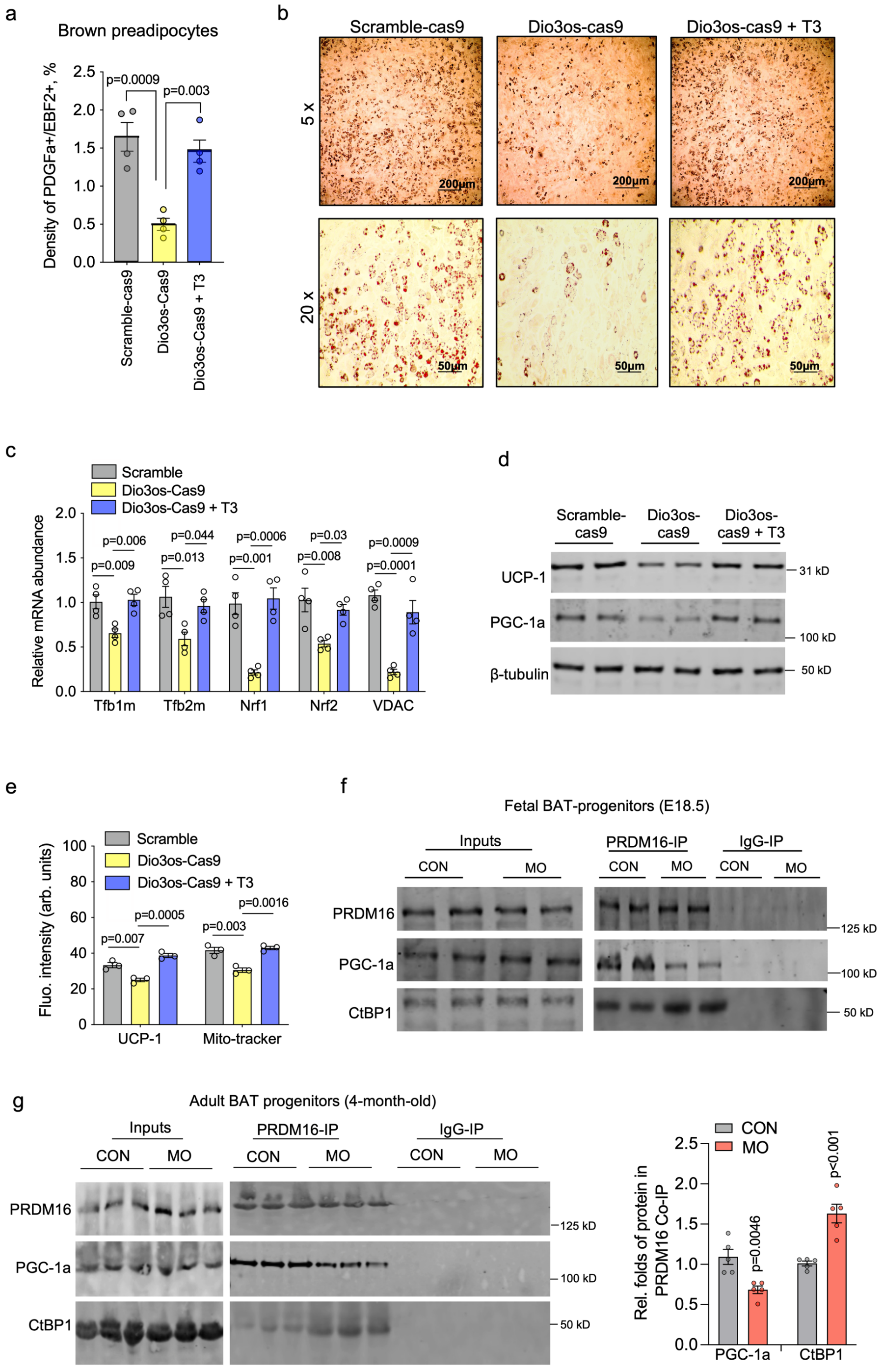

Supplementary Figure 8. *Dio3os* inactivation impairs brown adipogenesis and thermogenesis of mouse embryonic fibroblasts.

a. Quantified brown preadipocyte density (Lin: EBF2+/PDGFRa+) after inducing brown adiogenic commitment of mouse embryonic stem cells (n = 4). **b.** Oil Red-O staining of

differentiated brown adipocytes (*n* = 3). **c.** Expression of mitochondrial biogenic genes. mRNA expression was normalized to 18S rRNA (*n* = 4). **d.** Immunoblotting measuring protein contents of UCP-1 and PGC-1a in mature brown adipocytes (*n* = 4). β-tubulin was used as a loading control. **e.** Intensity of UCP-1 and mito-tracker measured by image-J (*n* = 3). **f, g.** PRDM16 immunoprecipitation for measuring PGC-1a and CtBP1 binding proteins in isolated brown preadipocytes from fetal (E18.5, f) (*n* = 6) and adult (4-month-old, g) BAT (*n* = 5). PRDM16, PGC-1a and CtBP1 were detected by immunoblotting. IgG was used as a negative control in immunoprecipitation. Data are presented as mean ± s.e.m. One-way ANOVA with adjusted multiple-comparison, and unpaired Student *t*-test with two tailed distribution were used in data analyses.

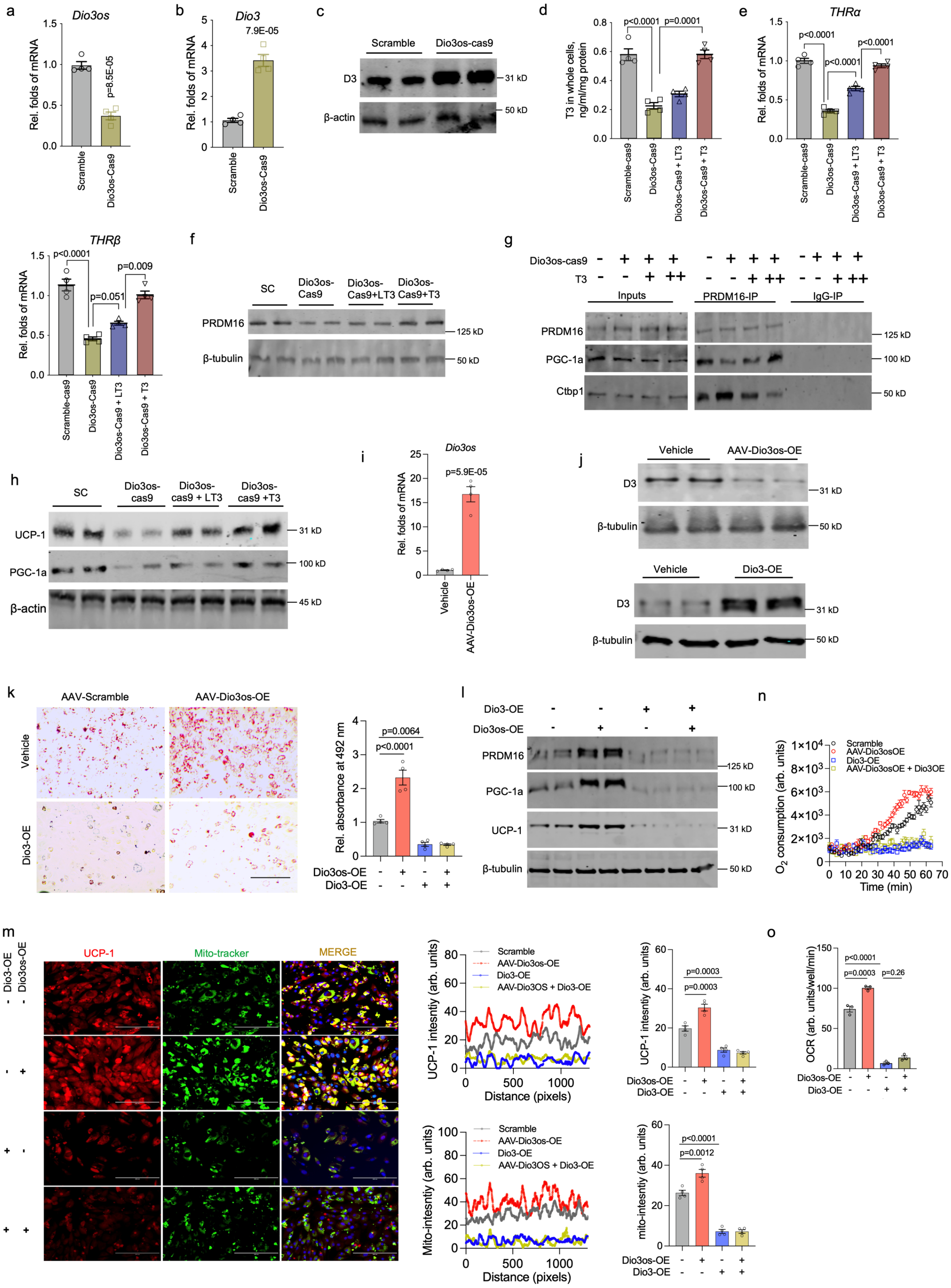

a
Dio3os
Rel. folds of mRNA
p=8.5E-05
Scramble
Dio3os-Cas9
b
Dio3
7.9E-05
Rel. folds of mRNA
Scramble
Dio3os-Cas9
c
Scramble
Dio3os-cas9
D3
31 kD
β-actin
50 kD
d
p<0.0001
p=0.0001
T3 in whole cells, ng/ml/mg protein
Scramble-cas9
Dio3os-Cas9
Dio3os-Cas9 + LT3
Dio3os-Cas9 + T3
e
THRα
p<0.0001
p<0.0001
p<0.0001
Rel. folds of mRNA
Scramble-cas9
Dio3os-Cas9
Dio3os-Cas9 + LT3
Dio3os-Cas9 + T3
THRβ
p<0.0001
p=0.009
p=0.051
Rel. folds of mRNA
Scramble-cas9
Dio3os-Cas9
Dio3os-Cas9 + LT3
Dio3os-Cas9 + T3
f
SC
Dio3os-Cas9
Dio3os-Cas9+LT3
Dio3os-Cas9+T3
PRDM16
125 kD
β-tubulin
50 kD
g
Dio3os-cas9
T3
Inputs
PRDM16-IP
IgG-IP
PRDM16
125 kD
PGC-1a
100 kD
Ctbp1
50 kD
h
SC
Dio3os-cas9
Dio3os-cas9 + LT3
Dio3os-cas9 +T3
UCP-1
31 kD
PGC-1a
100 kD
β-actin
45 kD
i
Dio3os
p=5.9E-05
Rel. folds of mRNA
Vehicle
AAV-Dio3os-OE
j
Vehicle
AAV-Dio3os-OE
D3
31 kD
β-tubulin
50 kD
Vehicle
Dio3-OE
D3
31 kD
β-tubulin
50 kD
k
AAV-Scramble
AAV-Dio3os-OE
Vehicle
Dio3-OE
p=0.0064
p<0.0001
Rel. absorbance at 492 nm
Dio3os-OE
Dio3-OE
l
Dio3-OE
Dio3os-OE
PRDM16
125 kD
PGC-1a
100 kD
UCP-1
31 kD
β-tubulin
50 kD
n
Scramble
AAV-Dio3osOE
Dio3-OE
AAV-Dio3osOE + Dio3OE
O_2 consumption (arb. units)
Time (min)
m
Dio3-OE
Dio3os-OE
UCP-1
Mito-tracker
MERGE
Scramble
AAV-Dio3os-OE
Dio3-OE
AAV-Dio3OS + Dio3-OE
UCP-1 intensity (arb. units)
Distance (pixels)
p=0.0003
p=0.0003
UCP-1 intensity (arb. units)
Dio3os-OE
Dio3-OE
Mito-intensity (arb. units)
Distance (pixels)
p<0.0001
p=0.0012
mito-intensity (arb. units)
o
p<0.0001
p=0.0003
p=0.26
OCR (arb. units/well/min)
Dio3os-OE
Dio3-OE

Supplementary Figure 9. *Dio3os* regulates brown adipogenesis via *Dio3*.

a. mRNA expression of *Dio3os* in spheroids after Dio3os-crRNA transfection (*n* = 4). Gene expression was normalized to 18S rRNA. **b, c.** mRNA and protein contents of D3 in spheroids after brown adipogenic induction (*n* = 4). β-actin was used as a loading control. **d, e.** During brown adipogenic commitment, T3 concentration (d) and mRNA expression of thyroid hormone receptors (e) in spheroids (*n* = 4). mRNA expression was normalized to 18S rRNA (5 days). **f.** After brown adipogenic commitment for 5 days, PRDM16 protein content was measured by immunoblotting (*n* = 3). β-tubulin was used as a loading control. **g.** After brown adipogenic commitment (5 days), immunoprecipitation was used for measuring PRDM16 interaction with PGC-1a or CtBP1 protein (*n* = 3). IgG was used as a negative control. **h.** Protein abundance of UCP-1 and PGC-1a in differentiated brown adipocytes (*n* = 3). β-actin was used as a loading control. **i-o.** Mouse embryonic fibroblasts (MEFs) were transfected with scramble, or AAV8-Dio3os-CMV viral particles (AAV-Dio3os-OE), or *Dio3* open reading frame (ORF) plasmid (Dio3-OE), or both followed brown adipocyte induction for 5 days. **i.** mRNA expression of *Dio3os* in MEF transfected with AAV-Dio3os (*n* = 4). mRNA expression was normalized to 18S rRNA. **j.** Immunoblotting measuring D3 protein in MEFs transfected with AAV-Dio3os or Dio3-ORF plasmid. β-tubulin was used as a loading control (*n* = 4). **k.** Oil-Red O staining in differentiated brown adipocytes (*n* = 4). Lipids were quantified by a microplate reader at 492 nm (*n* = 4). **l.** Immunoblotting measurements of PRDM16, PGC-1a and UCP-1 proteins in differentiated brown adipocytes (*n* = 4). β-tubulin was used as a loading control. **m.** Immunostaining of UCP-1 and mitochondria in differentiated brown adipocytes. Intensity was quantified by image-J (*n* = 4). **n, o.** Extracellular oxygen consumption (n) and OCR (o) of differentiated brown adipocytes measured by fluorescent assays (*n* = 3). Data are presented as

mean ± s.e.m. One-way ANOVA with adjusted multiple-comparison, and unpaired Student t-test with two tailed distribution were used in data analyses.

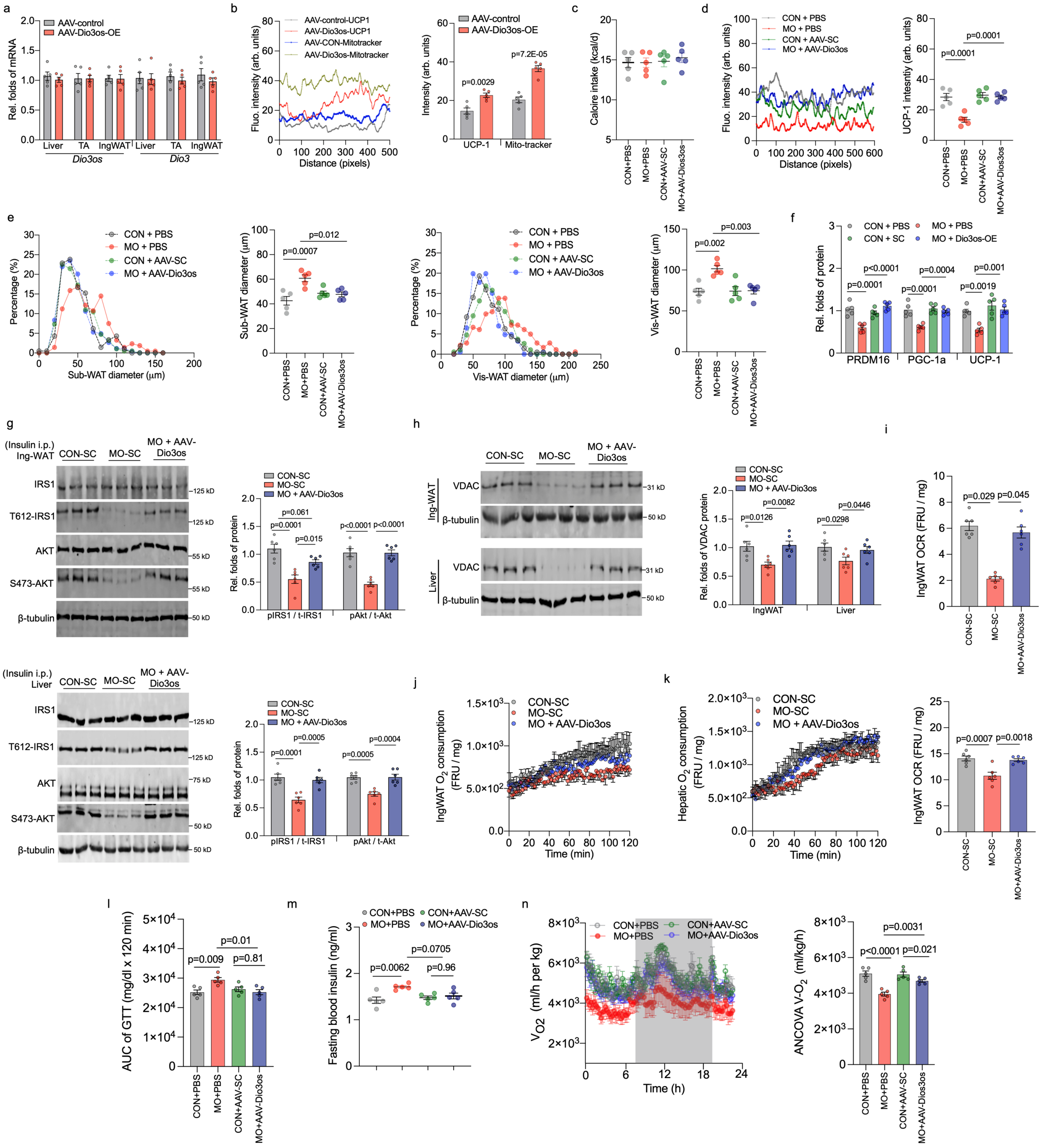

Supplementary Figure 10. ***Dio3os*** **activation in brown fat rescues impaired insulin signaling and mitochondrial respiration in liver and WAT of MO female offspring.**

a. *Dio3os* and *Dio3* mRNA expression in liver, tibialis anterior muscle (TA) and inguinal WAT in mice with scramble or AAV-Dio3os administration in BAT (n = 5). **b.** Fluorescent intensity of

UCP-1 and mitochondira in BAT (*n* = 5). **c.** Caloire intake of MO offspring received scramble or AAV-Dio3os (*n* = 5). **d, e.** Intensity of UCP-1 and mitochondrial flurorescence in BAT, and inguinal- and gonadal adipocyte size measured by image-J. **f.** Quantified protein contents of PRDM16, PGC-1a and UCP-1 in offspring BAT (*n* = 5). **g.** Immunoblotting measuring IRS-1 (T612) and AKT (S473) phosphorylation in offspring inguinal WAT and liver stimulated by intraperitoneal insulin administration (1 U/kg) after 30 min (*n* = 5). β-tubulin was as a loading control. **h.** Immunoblotting measuring VDAC protein in inguinal-WAT and liver of offspring administered with scramble or AAV-Dio3os in BAT (*n* = 6). **i-k.** Fluorescent assays measuring extracellular oxygen consumption rate (OCR) in offspring inguinal WAT and liver *ex-vivo* (*n* = 6). Tissues were freshly prepared and cultured in DMEM at 37˚C, and OCR was normalized to tissue mass. **l.** Quantified glucose tolerance test (area under curve) in MO offspring with AAV-Dio3os administration (*n* = 5). **m.** Fasting insulin concentration in serum (*n* = 5). **n.** Oxygen consumption at 5-week post-injection (*n* = 5). Metabolic data were normalized to total body mass according to guideline of NIDDK MCCP ANOVA tool. Data are mean ± s.e.m. Each pregnancy (dam) was used as a replicate unit; unpaired Student's *t*-test with two-tailed distribution, and one-way ANOVA with adjusted multiple-comparison were used in statistical analyses.

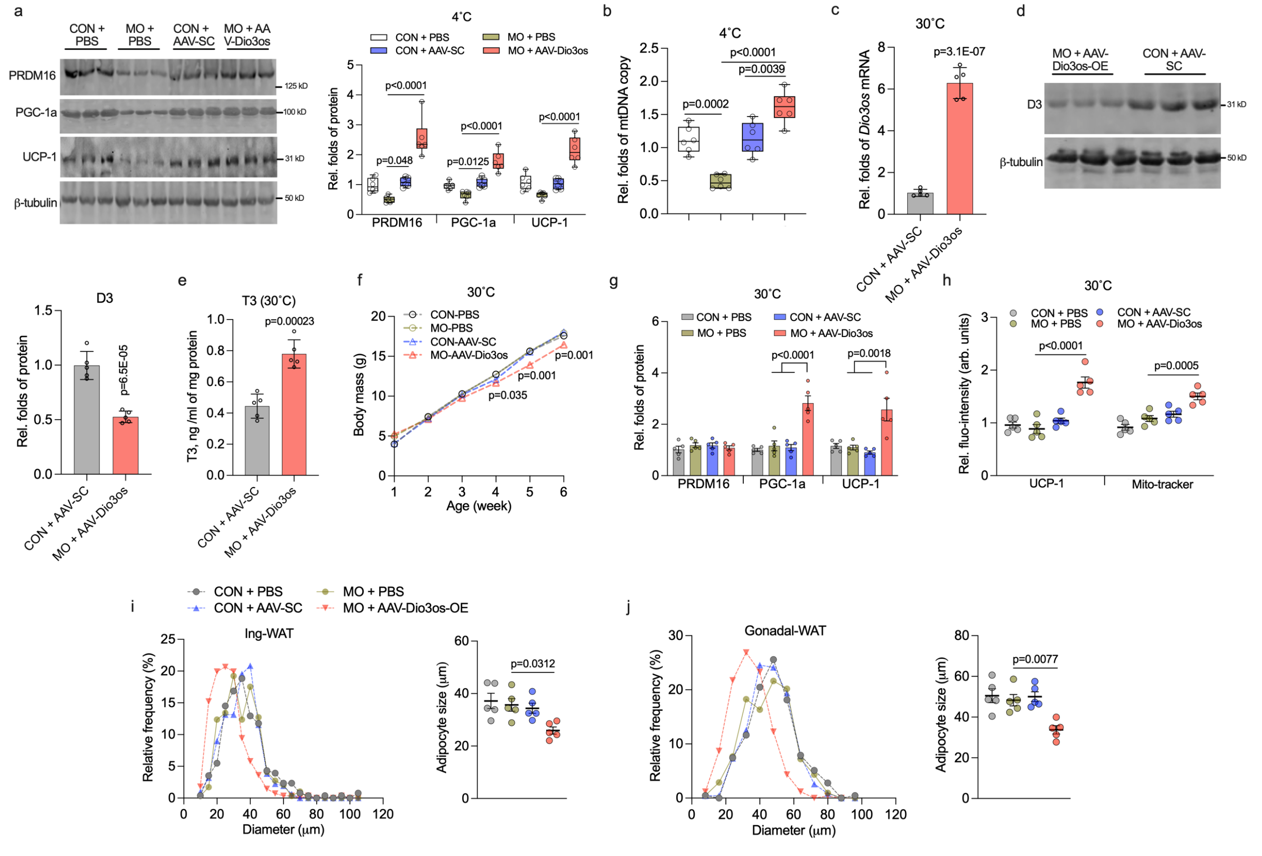

Supplementary Figure 11. ***Dio3os* activation in BAT improves cold resistance and glucose sensitivity of MO offspring at 4˚C and 30˚C.**

a. Immunoblotting measuring PRDM16, PGC-1a and UCP-1 proteins in BAT after 4˚C exposure (*n* = 6). β-Tubulin was as a loading control. (*n* = 6). Whisker of box plots shows mean and individual values from minimum to maximum. **b.** Mitochondrial DNA (mtDNA) copy number in BAT after 4˚C exposure (*n* = 6). Whisker of box plots shows mean and individual values from minimum to maximum. **c.** The mRNA expression of *Dio3os* in offspring BAT administered with scramble or AAV-Dio3os at 30˚C. Expression was normalized to 18S rRNA (*n* = 5). **d.** Immunoblotting measuring Dio3 protein in offspring BAT at 30˚C (*n* = 5). β-Tubulin was as a loading control. **e.** T3 concentration in offspring BAT at 30˚C (*n* = 5). **f.** Body mass at 30˚C (*n* = 5). **g.** Quantification of PRDM16, UCP-1 and PGC-1a proteins in BAT at 30˚C (*n* = 5). **h-j.** Quantification of UCP-1, mitochondrial fluorescent intensity (h), and diameter of inguinal (i) and

gonadal white adipocytes (j) using image-J (n = 5). Each pregnancy (dam) was used as a replicate unit; unpaired Student's t-test with two-tailed distribution, and two-way ANOVA with Bonferroni post hoc analysis were used in data analyses.

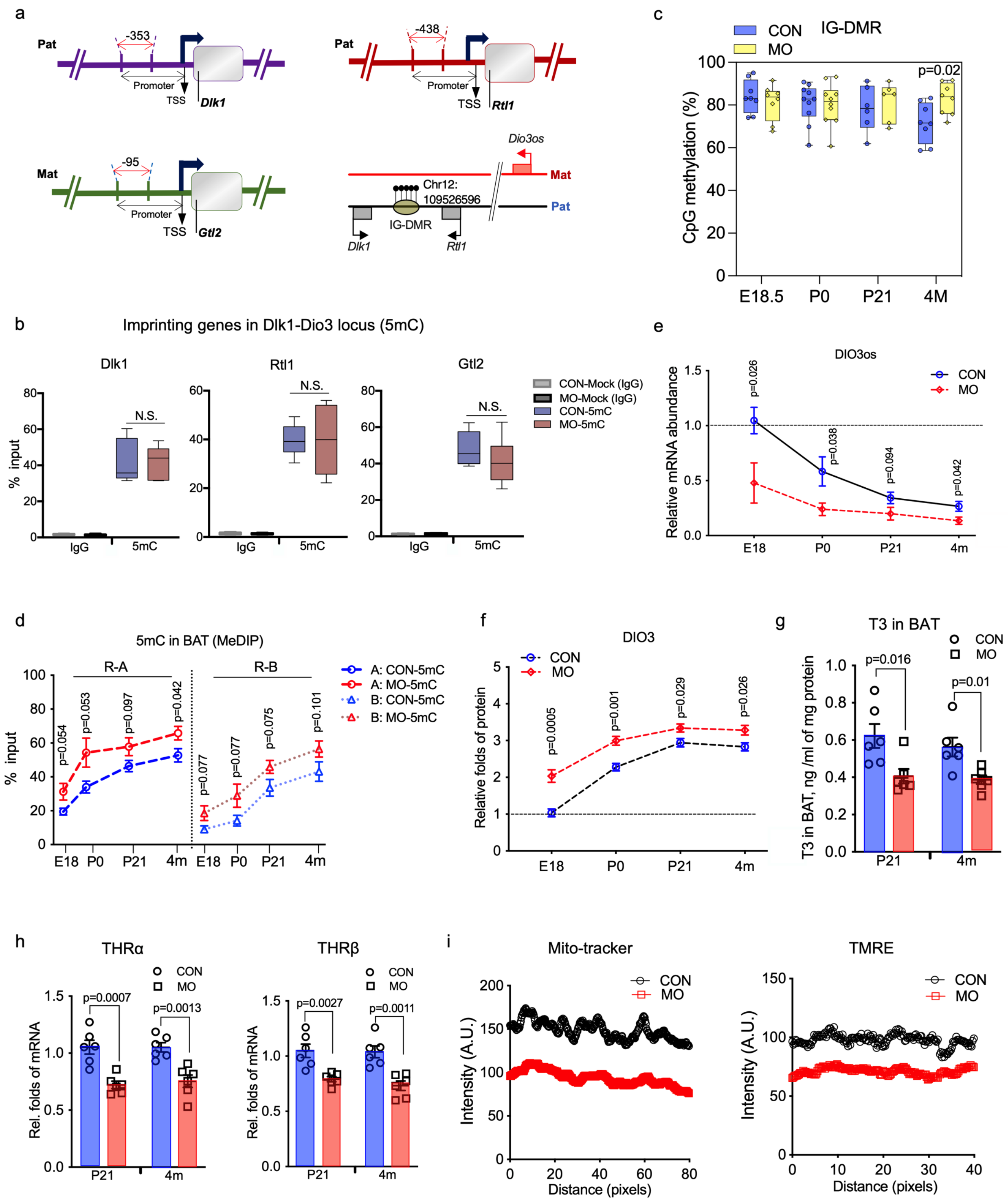

Supplementary Figure 12. Maternal obesity increases DNA methylation in the *Dio3os* promoter in offspring brown fat.

a. Diagrams shows CpG island in the promoter regions of paternal imprinting genes *Dlk1*, *Rtl1* and maternal imprinting gene *Gtl2*. Bisulfite pyrosequencing assayed percentage of CpG methylation in IG-DMR. **b.** Abundance of 5-methyl cytosine (5mC) quantified by MeDIP-qPCR in proximal promoters of imprinted genes in fetal BAT (E18). Mock IgG was used as a negative control (n = 6). Whisker of box plots shows mean and minimum to maximum values. **c.** Bisulfite pyrosequencing measurement of CpG percentage in IG-DMR in offspring BAT from fetuses to adults (n = 8 at E18.5, n = 10 at P0, n = 6 at P21, n = 8 at 4-month-old). Whiskers of plot box show mean and individual values from minimum to maximum. **d.** DNA methylation in *Dio3os* proximal CpG rich region R-A, and promoter region R-B in offspring BAT quantified by MeDIP-qPCR (n = 6). Mock IgG was used as a negative control. **e**, **f**. *Dio3os* (e) and *Dio3* (f) expression in offspring BAT (n = 6). The mRNA expression was normalized to 18S rRNA and protein expression was normalized to β-tubulin. **g.** T3 concentration in BAT of female offspring at weaning (P21) and 4 months of age (n = 6). **h.** The mRNA expression of thyroid hormone receptors in female offspring BAT at weaning and 4 months of age (n = 6). **i.** Quantified intensity of mitochondrial density (mito-tracker) and membrane potentials (TMRE) in control and obese dam oocytes using ImageJ (4 oocytes, n = 6 mice). Data are mean ± s.e.m. Each pregnancy (dam) was as a replicate unit; unpaired Student's t-test with two-tailed distribution was used in statistical analyses.

Supplementary Table 1. **List of primer sequences for gene expression and genome editing analyses**

Name	Forward primers	Reverse primers
RT-PCR		
18s	GTAACCCGTTGAACCCCATT	CCATCCAATCGGTAGTAGCG
Ucp-1	ACTGCCACACCTCCAGTCATT	CTTTGCCTCACTCAGGATTGG
Prdm16	CAGCACGGTGAAGCCATTC	GCGTGCATCCGCTTGTG
Ppargc1a	CCATACACAACCGCAGTCGC	GTGGGAGGAGTTAGGCCTGC
Cidea	ATCACAACTGGCCTGGTTACG	TACTACCCGGTGTCCATTTCT
Elvol3	TCCGCGTTCTCATGTAGGTCT	GGACCTGATCCAACCCTATGA
Pparg	AAACTCTGGGAGATTCTCCTGTT	GCATCTCTGTGTCAACCATGGT
C/EBPa	TGCGCAAGAGCCGAGATAAA	CCTTCTGTTGCGTCTCCACG
FASN	GGAGGTGGTGATAGCCGGTAT	TGGGTAATCCATAGAGCCCAG
Cirbp	CTTTTTCCGTGGGGGACGAA	TCGTTGTGTGTAGCATAACTGTCA
Dazap2	CACCATGAACAGCAAAGGTCAA	TGGGGATTGTGGAGCCTAGA
Hsbp1	ATCACTGGCAAGCACGAAGA	GGCCTCGAAAGTAACCGGAA
Hsp70	CCGACAAGGAGGAGTTCGTG	GACAGTCCTCAAGGCCACAT
p21	ATCCAGACATTCAGAGCCACAG	GACCCAGGGCTCAGGTAGAC
p53	GTTCCGGGAGCTGAATGAGG	TTTTATGGCGGGAAGTAGAC
Dio2	ATTATGCCTCGGAGAAGA	ACCAAAGTTGACCACCAG
Dio3	CAGTGAAGGCGAGGAGATG	CTTGTGCGTAGTCGAGGAT
Dio3os	CATGTGGCAGCCATAGTCT	CAGGGTTTCCCAAGTGTC
Thra	TGCTAATGTCAACAGACCGCT	GCTGCCCCCTTGTACAGAAT
Thrb	CGTGTTTTCCCTCTCGTCCA	GGCAGGCTTCAGACATTCCT
Mcp8	TGCCCTTGGTTACTTCGTCC	CAGGAATGAGAGGACCTGCAA
Dlk1	ATCAATGGTTCTCCCTGCCA	CACTGGCGCAGTTGCTCA
Rtl1	GGCGCTAACAGTGGTTTTGG	TCAACGGTGTTGGATGAGCC
Zfp423	GTCACCAGTGCCCAGGAAGAAGAC	AACATCTGGTTGCACAGTTTACACTCAT
Slc7a10	GGAGTCACTATCCTGGGCCT	AGCGTGTCATGGACTCTGTG
AP2	CGACAGGAAGGTGAAGAGCATCATA	CATAAACTCTTGTGGAAGTCACGCCT
Glut4	CTAGGCATCAATGCTGTTTTCTA	CGAGACCAACGTGAAGACCGTATT
IL6	CACTTCACAAGTCGGAGGCTTA	GCAAGTGCATCATCGTTGTTC
IL1a	TCTGCCATTGACCATCTC	ATCTTCCCGTTGCTTGAC
IL1b	GTTCCCATTAGACAACTGC	GATTCTTTCCTTTGAGGC
IP10	CAGTGAGAATGAGGGCCATAGG	CGGATTCAGACATCTCTGCTCAT

TNFa	CATCTTCTCAAAATTCGAGTGACAA	TGGGAGTAGACAAGGTACAACCC
Tfb1m	CACCGAGGGCTTGGAATGTT	TAGAACCCGCAGCTTTCTGG
Tfb2m	TAAAGCTGGTGCCAGAGTGG	AGGAACACCTGCTGACCAAG
Nrf1	ACGTTACAGGGCGGTGAAAT	ATCTGGACCAGGCCATTAGC
Nrf2	GCCCTCAGCATGATGGACTT	TGGTGTCTGTCTGGATGTGC
VDAC	CTCCGCCGAGAGGACGAA	CTCCCTATGGGGTCTCGCTC
Gtl2	GATGACATCATCGGCTCACAC	CTAGCTGGCCACTGATGATCC
PolyA method RT-PCR		
miR-1247-5p	ACCCGTCCCGTTCGT	
miR-1247-3p	CGGGAACGTCGAGACTG	
miR-379-5p	TGGTAGACTATGGAACGTAGG	
miR-379-3p	TATGTAACATGGTCCACTAACT	
miR-411-5p	TAGTAGACCGTATAGCGTACG	
miR-411-3p	TATGTAACACGGTCCACTAACC	
miR-493-5p	TTGTACATGGTAGGCTTTC	
miR-493-3p	TGAAGGTCCTACTGTGTGC	
miR-485-5p	AGAGGCTGGCCGTGAT	
miR-485-3p	AGTCATACACGGCTCTCCTC	
miR-654-5p	TGGTAAGCTGCAGAACATGTGT	
miR-654-3p	TATGTCTGCTGACCATCACCTT	
crRNA for CIRSPR-Cas9		
Dio3os upstream	CCGGACGTTGCTCTCTGCCC	
Dio3os downstream	CCTTGGAGAGACTTCTGGGA	
Scramble-1	TGGGTCCTAAACGCGGTTCA	
Scramble-2	TGAGCGCTTTCCCGATCCGG	
PCR primers for MeDIP PCR		
Dio3os-A	TAGCTGTTGCCTGAACGCGG	AGTTCGCGAAGGTGGCTCAG
Dio3os-B	GCGAGGAGGCAACTTTGGGT	CAGCTGACCCGGGAACCG
Dio3os-C	AAATCTTGGGTCGAGGGCGG	TGGGACCTAGGAGGTGCGAG
Dlk1	GCTTCTGACCCGCGCCATGT	CAGGAACCAAGCCAGGCGGG
Rtl1	ACCACCAGAAGGCCAGGGCA	TGGCCCATGGTGTCAGGGCT
Gtl2	TCCCCCTCCTCCACCCACCT	AAGAGCCCCTACCTGGCGCA
