# Supplementary material for: Imprinted lncRNA Dio3os preprograms intergenerational brown fat development and obesity resistance
Source: Nat Commun. 2021 Nov 25;12:6845. doi: 10.1038/s41467-021-27171-1 (PMC8617289; doi:10.1038/s41467-021-27171-1)
Supplement: Supplementary file 3 — Reporting Summary [file 41467_2021_27171_MOESM3_ESM.pdf]

nature portfolio

Corresponding author(s): Min Du

Last updated by author(s): Oct 28, 2021

Reporting Summary

Nature Portfolio wishes to improve the reproducibility of the work that we publish. This form provides structure for consistency and transparency in reporting. For further information on Nature Portfolio policies, see our Editorial Policies and the Editorial Policy Checklist.

Statistics

For all statistical analyses, confirm that the following items are present in the figure legend, table legend, main text, or Methods section.

n/a	Confirmed	
☐	☒	The exact sample size (*n*) for each experimental group/condition, given as a discrete number and unit of measurement
☐	☒	A statement on whether measurements were taken from distinct samples or whether the same sample was measured repeatedly
☐	☒	The statistical test(s) used AND whether they are one- or two-sided<br>*Only common tests should be described solely by name; describe more complex techniques in the Methods section.*
☐	☒	A description of all covariates tested
☐	☒	A description of any assumptions or corrections, such as tests of normality and adjustment for multiple comparisons
☐	☒	A full description of the statistical parameters including central tendency (e.g. means) or other basic estimates (e.g. regression coefficient) AND variation (e.g. standard deviation) or associated estimates of uncertainty (e.g. confidence intervals)
☐	☒	For null hypothesis testing, the test statistic (e.g. F, t, r) with confidence intervals, effect sizes, degrees of freedom and P value noted<br>*Give P values as exact values whenever suitable.*
☒	☐	For Bayesian analysis, information on the choice of priors and Markov chain Monte Carlo settings
☒	☐	For hierarchical and complex designs, identification of the appropriate level for tests and full reporting of outcomes
☐	☒	Estimates of effect sizes (e.g. Cohen's d, Pearson's r), indicating how they were calculated

Our web collection on statistics for biologists contains articles on many of the points above.

Software and code

Policy information about availability of computer code

Data collection	For western blot band intensity analysis, data were collected by Image Studio Lite 5.2 (Licor Biosciences); for qPCR ct value analysis, data were collected by BioRad CFX manager 3.1 software (CFX Connect); For indirect calorimetry measurements, data were collected by Oxymax v 4.93 software (Columbus Instruments); for measuring T3/T4/TSH/adrenaline/noradrenaline concentrations, data were collected by Gen5 2.01 software (BioTek Synergy H1); for flow cytometry analysis, data were collected by FlowJo Software Version 10; for surface temperature analysis, data were collected by FLIR-Tools-software (FLIR system).
Data analysis	Graph Pad version 7<br>SAS version 9.4<br>ImageJ 2.1.0

For manuscripts utilizing custom algorithms or software that are central to the research but not yet described in published literature, software must be made available to editors and reviewers. We strongly encourage code deposition in a community repository (e.g. GitHub). See the Nature Portfolio guidelines for submitting code & software for further information.

Data

Policy information about availability of data

All manuscripts must include a data availability statement. This statement should provide the following information, where applicable:

- Accession codes, unique identifiers, or web links for publicly available datasets
- A description of any restrictions on data availability
- For clinical datasets or third party data, please ensure that the statement adheres to our policy

The authors declare that the data supporting the findings of this study are available within the paper and its supplementary information files. Source data are

provided as a Source Data file.

Field-specific reporting

Please select the one below that is the best fit for your research. If you are not sure, read the appropriate sections before making your selection.

- [x] Life sciences
- [ ] Behavioural & social sciences
- [ ] Ecological, evolutionary & environmental sciences

For a reference copy of the document with all sections, see nature.com/documents/nr-reporting-summary-flat.pdf

Life sciences study design

All studies must disclose on these points even when the disclosure is negative.

Sample size	Sample size of mice (for offspring body weight and fat mass change) was determined by mean and standard deviation obtained in our previous studies for maternal high fat feeding trials (Scientific Reports 2016, 6:34345; Journal of Physiology 2016, 594(15):4453-66). Statistic power calculations were set using a= 0.05, desired power = 0.8. Sample size was reported in individual figure legends.
Data exclusions	No data exclusions
Replication	We performed experiments at least twice independently. For some experiments, we replicated more than 2 times. Replications were successful.
Randomization	In trials, female mice with similar body weight and age were randomly assigned into dietary treatments (HFD vs control) before mating. Offspring from each treatment were also randomly assigned into diets (control and HFD), drugs or AAV administration. In measuring energy expenditure, animals were also randomly assigned into individual chambers. In cell culture studies, cells were also randomly assigned into individual treatments.
Blinding	During sample collection and measuring, including tissue collection and qPCR analysis, investigators were blinded to treatments. For histological and electronic microscopy, investigators were blinded to dietary and drug treatments. For energy expenditure, glucose and insulin tolerance tests, observers were all blinded to both dietary and AAV treatments. In cell culture studies, investigators need to distinguish labeling and perform the cell treatment, thus, blinding was not possible. Investigators were blinded to group allocation during data collection and analysis for other experiments.

Reporting for specific materials, systems and methods

We require information from authors about some types of materials, experimental systems and methods used in many studies. Here, indicate whether each material, system or method listed is relevant to your study. If you are not sure if a list item applies to your research, read the appropriate section before selecting a response.

Materials & experimental systems

n/a	Involved in the study	
[ ]	[x]	Antibodies
[ ]	[x]	Eukaryotic cell lines
[x]	[ ]	Palaeontology and archaeology
[ ]	[x]	Animals and other organisms
[x]	[ ]	Human research participants
[x]	[ ]	Clinical data
[x]	[ ]	Dual use research of concern

Methods

n/a	Involved in the study	
[x]	[ ]	ChIP-seq
[ ]	[x]	Flow cytometry
[x]	[ ]	MRI-based neuroimaging

Antibodies

Antibodies used	UCP-1 (#14670; Cell Signaling); PRDM16 (PA5-20872; Thermo Fisher Scientific);PGC-1a (#66369-I; Proteintech); Dio2 (ab77481; Abcam);Dio3 (ab233035; Abcam); VDAC (#4866; Cell Signaling); CtBP1 (ab14411; Abcam); TH (25859-1-AP; ProteinTech); β-actin (MA5-15739; Thermo Fisher Scientific); β-tubulin (#179513; Abcam); PDGFRα APC (1:200) (#135907; Biolegend);EBF2 (BS-11740R; Bioss, MA); Alex Fluor-anti-rabbit-488 (#100343867; Biolegend);MitoSpy-green (#424805; Biolegend);CTB (#C34775, Thermo Fisher);TMRE-red (#115532; Sigma); 5-methylcytosine (5mC) (# A3002; ZYMO Research); IgG (#31430; Thermo); anti-mouse IRDye 680 (C80926-17; LI-COR); anti-rabbit IRDye 800cw (C70918-03; LI-COR)
Validation	The validation of antibodies used in the study includes:<br>UCP1 (validated in publication Diabetes 2020 Aug; 69(8): 1662-1674)<br>PGC-1a (validated in publication DOI: 10.1126/sciadv.aaz0359)<br>Dio2 (validated in publication Nat Med 24:39-49 2018)<br>Dio3 (validated in https://www.abcam.com/dio3-antibody-ab233035.html)<br>VDAC (validated in publication Diabetes 2020 Aug; 69(8): 1662-1674)

CtBP1 (validated in publication doi: 10.18632/oncotarget.26157)
TH (validated in publication Diabetes 2018 Feb; 67(2): 235-247)
β-actin (validated in https://www.thermofisher.com/antibody/product/beta-Actin-Loading-Control-Antibody-clone-BA3R-Monoclonal/MA5-15739)
PDGFa APC (validated in publication https://doi.org/10.1073/pnas.1412685111)
EBF2 (validated in publication https://doi.org/10.1038/s41467-020-18865-z)
PRDM16 (https://doi.org/10.1016/j.cmet.2016.08.010)
MitoSpy green (validated in publication Diabetes 2020 Aug; 69(8): 1662-1674)
CTB (validated in publication Science (2013) 339:1290-1295)
TMRE (validated in https://www.sigmaaldrich.com/US/en/product/sigma/87917)
β-tubulin (validated in publication https://doi.org/10.1016/j.cmet.2016.08.010)
5mC (https://doi.org/10.1016/j.cmet.2016.08.010)
Alex Fluor 488 (https://doi.org/10.1016/j.cmet.2016.08.010)
Anti-mouse IgG (validated in publication https://doi.org/10.1016/j.cmet.2016.08.010)
Anti-mouse IRDye 680 (validated in publication Diabetes 2020 Aug; 69(8): 1662-1674)
Anti-rabbit IRDye 800CW (validated in publication Diabetes 2020 Aug; 69(8): 1662-1674)

Eukaryotic cell lines

Policy information about cell lines

Cell line source(s)	mouse embryonic fibroblasts (MEFs); brown fat stromal vascular fractions (SVFs); AAV-293 cell lines (Agilent, CA, USA)
Authentication	MEFs and SVFs were isolated from C57BL/6J mice following previous protocols (https://doi.org/10.1016/j.cmet.2016.08.010; Diabetes 2020 Aug; 69(8): 1662-1674), AAV-293 cells were purchased from Agilent.
Mycoplasma contamination	All cells were tested for mycoplasma contamination with negative results.
Commonly misidentified lines (See ICLAC register)	No commonly misidentified cell lines were used in this study.

Animals and other organisms

Policy information about studies involving animals; ARRIVE guidelines recommended for reporting animal research

Laboratory animals	Mouse: C57BL/6J (Jackson Lab, Bar Harbor, ME, USA). 8-wk of age female mice were fed control diet (D12450H, Research Diets) or high fat diet (D12451) for 10 weeks before mating, and further fed with their respective diets until weaning at 22˚C, 12-hour light/12-hour dark and 50% humidity. After weaning, both male and female offspring from each treatment were further assigned to either control or obesogenic diet (D12492) for 12 weeks. In addition, mouse offspring was also subjected to 30˚C for 4 weeks or 4˚C for 4 days to test BAT independent effects.
Wild animals	Study did not involve wild animals.
Field-collected samples	Study did not involve field collected samples.
Ethics oversight	All animal experiments were conducted according to the protocol approved by the Institute of Animal Care and Use Committee (IACUC) at Washington State University (WSU, WA, USA). The animal facility is accredited by the Association for Assessment and Accreditation of Laboratory Animal Care (AAALAC).

Note that full information on the approval of the study protocol must also be provided in the manuscript.

Flow Cytometry

Plots

Confirm that:

- [x] The axis labels state the marker and fluorochrome used (e.g. CD4-FITC).
- [x] The axis scales are clearly visible. Include numbers along axes only for bottom left plot of group (a 'group' is an analysis of identical markers).
- [x] All plots are contour plots with outliers or pseudocolor plots.
- [x] A numerical value for number of cells or percentage (with statistics) is provided.

Methodology

Sample preparation	BAT primary stromal vascular fractions (SVFs) were freshly prepared and fixed in 4% PFA for 15 min. Fixed cells were slowly permeabilized with 100% methanol for 15 min on ice. Washed cells were blocked in 1 : 300 conjugated PDGFRα APC (1:200) (#135907; Biolegend, USA) and EBF2 anti-rabbit antibody 1 : 200 (#bs-11740R; Bioss, MA, USA) at room temperature for 2 h. Cells were further incubated in Alex Fluor-anti-rabbit-488 (#100343867; Biolegend) for 1 h and counted in a Sony SY3200 sorter (Sony, CA, USA).<br>For isolating SVFs, BAT was dissected and digested in DMEM FBS free medium containing 0.75 U/ml collagenase D (Roche,

	Pleasanton, CA) for 30 min in a shaker at 37˚C. Lysate was filtered through 100 and 40 µm strainers. Cells were collected by centrifuged at 500 × g for 5 min.
Instrument	Sony SY3200 Sorter
Software	Flowjo
Cell population abundance	Isolated SVFs were analyzed by individual mouse, and data were shown as scattering plots and reported as percentage in the Result Section.
Gating strategy	Gating schematic are included in Figure 3b and Figure 4i.

☒ Tick this box to confirm that a figure exemplifying the gating strategy is provided in the Supplementary Information.
